# Segregation Behavior of Polysaccharide–Polysaccharide Mixtures—A Feasibility Study

**DOI:** 10.3390/gels5020026

**Published:** 2019-05-13

**Authors:** Benjamin Zeeb, Theresa Jost, David Julian McClements, Jochen Weiss

**Affiliations:** 1 Department of Food Physics and Meat Science, Institute of Food Science and Biotechnology, University of Hohenheim, Garbenstrasse 21/25, 70599 Stuttgart, Germany; 2 Department of Food Science, University of Massachusetts, Amherst, MA 01003, USA

**Keywords:** segregation, association, biopolymer complexes, alginate, sugar beet pectin

## Abstract

The segregative phase separation behavior of biopolymer mixtures composed entirely of polysaccharides was investigated. First, the electrical, optical, and rheological properties of alginate, modified beet pectin, and unmodified beet pectin solutions were characterized to determine their electrical charge, molecular weight, solubility, and flow behavior. Second, suitable conditions for inducing phase segregation in biopolymer mixtures were established by measuring biopolymer concentrations and segregation times. Third, alginate–beet pectin mixtures were blended at pH 7 to promote segregation and the partitioning of the biopolymers between the upper and lower phases was determined using UV–visible spectrophotometry, colorimetry, and calcium sensitivity measurements. The results revealed that phase separation depended on the overall biopolymer concentration and the degree of biopolymer hydrophobicity. A two-phase system could be formed when modified beet pectins (DE 68%) were used but not when unmodified ones (DE 53%) were used. Our measurements demonstrated that the phase separated systems consisted of a pectin-rich lower phase and an alginate-rich upper phase. These results suggest that novel structures may be formed by utilization of polysaccharide–polysaccharide phase separation. By controlling the product formulation and processing conditions it may therefore be possible to fabricate biopolymer particles with specific dimensions, shapes, and internal structures.

## 1. Introduction

Processed food products often contain a number of different food biopolymers that can interact with each other through a variety of different forces, which leads to a diverse range of microstructural organizations and physicochemical properties [1,2,3,4,5]. Careful manipulation of these interactions can therefore be used to create foods with novel or improved functional attributes [6,7]. In general, tunable microstructures can be created within biopolymer mixtures using either associative or segregative phase separation mechanisms [8,9,10]. Associative phase separation occurs when the different biopolymers are attracted to each other, whereas segregative phase separation occurs when they are repelled. The physicochemical properties of mixed biopolymer complexes is influenced by a combination of intrinsic and extrinsic parameters [11,12,13], such as molecular weight, charge, flexibility, hydrophobicity, pH, ionic strength, and temperature.

At sufficiently high concentrations, mixing two similarly charged or uncharged biopolymers may lead to phase separation due to restrictions in the freedom of orientation of the biopolymer molecules [9]. As a result, the mixture segregates into two phases with different compositions: one phase rich in Biopolymer A and depleted in Biopolymer B, and vice versa. The phase separated system can be converted into a ‘water-in-water’ emulsion by application of shear forces, leading to a suspension consisting of one aqueous phase dispersed as small droplets in another aqueous phase [14,15,16,17].

A substantial amount of research has been carried out to investigate segregative and associative phase separation phenomena in mixed biopolymer systems composed of proteins and polysaccharides [18,19,20]. In the present study, we were interested in studying segregative phase separation in mixed biopolymer systems consisting of polysaccharide–polysaccharide mixtures. In particular, we focused on the utilization of two forms of dietary fibers, since these may have health benefits when incorporated into food products. For this reason, we utilized two indigestible functional food polysaccharides: alginate (ALG) and sugar beet pectin (BP). In the case of beet pectin, we examined forms with various degrees of esterification so as to alter their hydrophobicity. It was hypothesized that both hydrophobic and electrostatic forces would be involved in the demixing process, leading to segregative phase separation. For the sake of comparison, a protein–polysaccharide mixture composed of caseinate (CAS) and alginate was used as a reference system.

## 2. Results and Discussion

### 2.1. Characterization of Biopolymer Properties

The purpose of these studies was to investigate the phase separation behavior of biopolymers mixtures entirely composed of polysaccharides induced by repulsive segregation (Table 1). To understand the factors impacting the tendency for phase separation to occur it is important to have information about the properties of the biopolymers used. For this reason, the molecular and physicochemical attributes of the various biopolymers used in this study were initially characterized. As such, ζ-potential, turbidity, and viscosity measurements were used to characterize the physicochemical properties of single caseinate, beet pectin, and alginate solutions.

**Figure 1:**
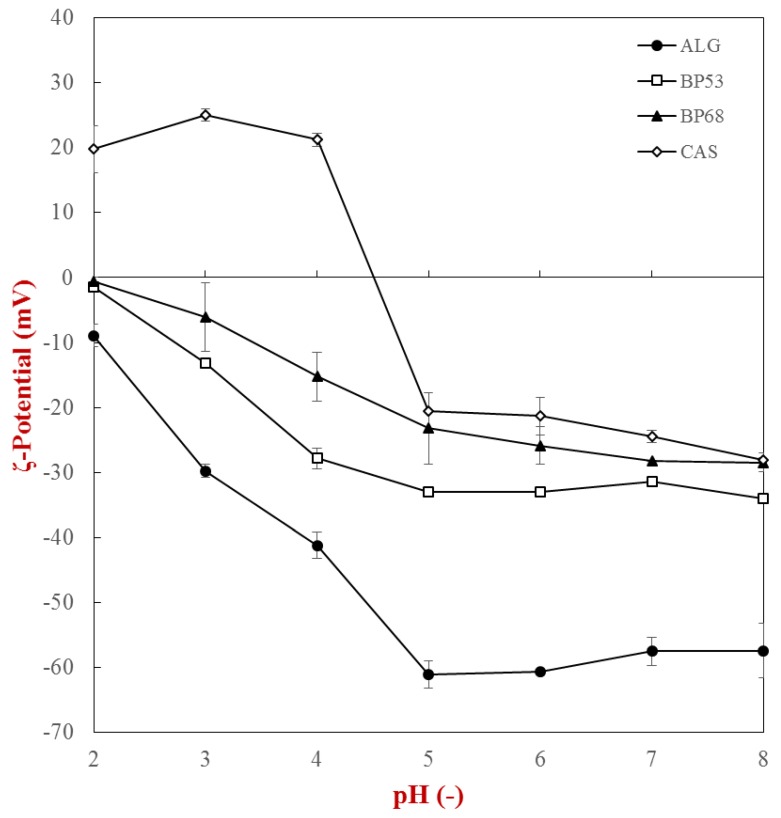
ζ-potential of single biopolymer solutions (*c_biopolymer_* = 0.1%) as a function of pH (2–8): caseinate (CAS), alginate (ALG), sugar beet pectin DE 53% (BP53), and sugar beet pectin DE 68% (BP68).

**Figure 2:**
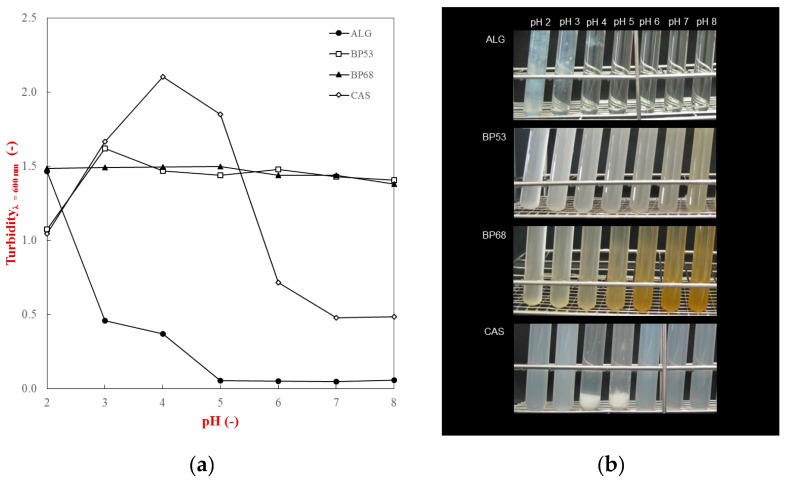
Turbidity (**a**) and phase separation (**b**) of single biopolymer solutions (*c_biopolymer_* = 0.1%) as a function of pH (2–8): caseinate (CAS), alginate (ALG), sugar beet pectin DE 53% (BP53), and sugar beet pectin DE 68% (BP68).

Initially, the influence of pH on the ζ-potential and turbidity of single biopolymers was determined (Figure 1 and Figure 2). In general, the biopolymers displayed the expected behavior when subjected to changes in solution pH. The anionic polysaccharides had a relatively high negative charge from pH 8 to 5, but the magnitude of their charge decreased as the pH was reduced from 5 to 2. This effect can be attributed to protonation of the carboxyl groups on the alginate and beet pectin molecules, which typically have pK_a_ values around 3.5. At neutral pH, the magnitude of the ζ–potential of the alginate molecules was considerably higher than that of the beet pectin molecules, which can be attributed to a higher linear charge density, i.e., more carboxyl groups per unit length of molecule. As expected, the negative charge on the beet pectin with the lower degree of esterification (53%) was greater than with the higher degree of esterification (68%), because it had more free carboxyl groups [4]. The ζ–potential of the caseinate went from negative to positive as the pH was decreased from 8 to 2, which can be attributed to changes in the charge status of the amino and carboxyl groups. The point of zero charge was around pH 4.5, which is close to the known isoelectric point of caseinate [21].

### 2.2. Precipiation and Sedimentation Behavior

Turbidity measurements were conducted to gain information about the pH-dependent phase separation of the individual biopolymers used in this study. After hydration in buffer solutions (pH 2 to 8), the turbidity of the biopolymer solutions was measured spectrophotometrically at 600 nm and photographic images taken to visualize any precipitation or sedimentation (Figure 2).

In general, the results showed that the pH had a significant effect on the precipitation and sedimentation behavior of the biopolymers. In particular, caseinate solutions strongly aggregated between pH 4 and 5, which led to an increase in turbidity and sediment formation. This phenomenon can be attributed to changes in the electrical characteristics of the casein with pH (Figure 1). The ζ-potential measurements showed that caseinate has isoelectric point (p*I*) around pH 4.5, with a strong negative or positive charge above or below this value. Consequently, there is a strong electrostatic repulsion between the molecules at pH values sufficiently above or below the p*I*, which inhibits protein aggregation and leads to a clear solution with no sedimentation [17]. Conversely, at the p*I*, the casein molecules aggregate with each other because the attractive interactions (van der Waals, hydrophobic attraction) outweigh the repulsive interactions (electrostatic and steric), leading to extensive precipitation and sedimentation. 

The pH-dependent solubility behavior of the polysaccharides was quite different from that of the protein. The sugar beet pectin solutions had relatively high and constant turbidity values across the entire pH range studied (Figure 2a). Visually, they had a quite cloudy appearance, but no sedimentation was observed at any pH value (Figure 2b). This result suggests that these sample may have contained some insoluble fragments from the sugar beet. The fact that these fragments were relatively stable to precipitation and sedimentation can be attributed to the strong electrostatic repulsion between them associated with their high negative charge (Figure 1). Interestingly, the beet pectin samples became increasingly yellowish when the pH was increased from 2 to 8. Previous studies have also suggested that the yellow and orange colors found in beet pectin samples may be due to the presence of natural pigments, such as betaxanthins [22,23]. The alginate samples exhibited quite different behavior. They remained optically clear from pH 8 to 4, but formed white clumps at lower pH follows (Figure 2b). This effect can be attributed to the steep reduction in the negative charge on the alginate molecules when the pH is reduced below 4 (Figure 1). Presumably, this effect was not observed for the beet pectin samples because they have long neutral side chains attached to the anionic backbone of the molecule, which should prevent the chains from coming too close together. In contrast, alginate is a linear molecule with no side-chains, so that once it loses its negative charge, the molecules can come into close contact through van der Waals and hydrogen bonding.

Based on these results, we selected pH 7 to investigate the segregation behavior of the mixed biopolymer systems due to the strong electrostatic repulsion without sedimentation in all of the samples.

### 2.3. Rheological Measurements

The purpose of this set of experiments was to determine the optimum concentration range that could be utilized to induce biopolymer segregation. Previous studies have shown that the segregation of a binary biopolymer mixture into two distinct phases is thermodynamically preferred when the total biopolymer concentration exceeds a critical level [24,25]. However, if the biopolymer concentration is too high, then the biopolymers may not separate because their movement is hindered by the formation of a highly viscous or gelled biopolymer matrix [26,27]. For this reason, the rheological properties of biopolymer solutions with different compositions were analyzed.

**Figure 3:**
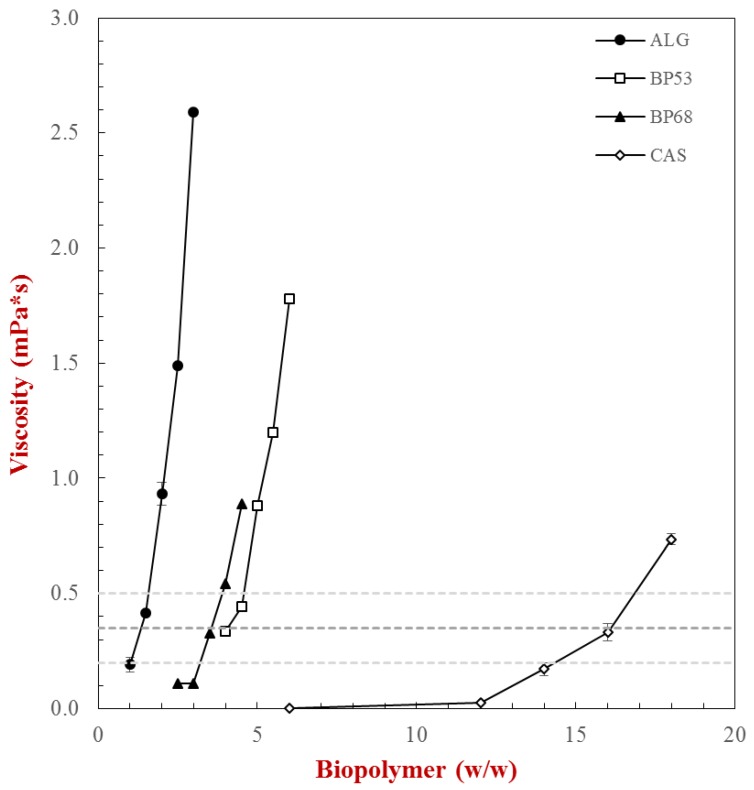
Impact of biopolymer concentration on the solution viscosity (measured at a shear rate = 100 s^−1^): caseinate (CAS), alginate (ALG), sugar beet pectin DE 53% (BP53), and sugar beet pectin DE 68% (BP68). The measurements were conducted at 25 °C.

Measurements of the shear viscosity versus shear rate profiles indicated a distinct shear thinning behavior for all the biopolymers tested (Figure S1). At a fixed shear rate (100 s^−1^) the shear viscosity versus biopolymer concentration profile of the biopolymers was distinctly different (Figure 3). The ability of the biopolymers to thicken the aqueous solutions increased in the following order: CAS < BP68 < BP53 < ALG. These differences in the thickening power of the biopolymers can be related to differences in their molecular structures [28].

To a first approximation, the ability of a biopolymer to increase the viscosity of an aqueous solution can be described by the following expressions [28]:

(1) $$\frac{\eta }{\eta_{1}}={\left(1-\frac{\phi_{E}}{\phi_{C}}\right)}^{-2}$$

(2) $$\phi_{E}=\frac{4}{3}\pi r_{H}^{3}\left(\frac{cN_{A}}{M}\right)$$

Here, *η* and *η*_1_ are the viscosities of the biopolymer solution and water, *Φ*_E_ is the effective volume fraction of the biopolymer molecules in solution (which is comprised of the volume occupied by the biopolymer chain as well as any entrained water), *Φ*_C_ is the critical packing fraction (which has a value where the biopolymer molecules become close packed into solution, i.e., about 0.57 ), *r*_H_ is the biopolymer hydrodynamic radius, *c* is the biopolymer concentration, *M* is the biopolymer molecular weight, and *N*_A_ is Avogadro’s number. These expressions indicate that the increase in viscosity or gelation of a biopolymer increases as its concentration and hydrodynamic radius increase.

Alginates are relatively large linear molecules that have a high radius of gyration, and therefore strong thickening power. In contrast, beet pectins are large branched molecules with a more compact structure and therefore lower radius of hydration. Finally, caseins are small flexible molecules and therefore have a still lower radius of hydration [21,29]. Based on the viscosity meaurements, we selected a maxium biopolymer concentration of 6% for the subsequent studies, as this led to solutions that were not too viscous and did not gel (Figure 3). As a result, these solutions should be suitable for allowing phase separation to occur in biopolymer mixtures.

### 2.4. Mixed Biopolymer Systems—Caseinate and Alginate

**Figure 4:**
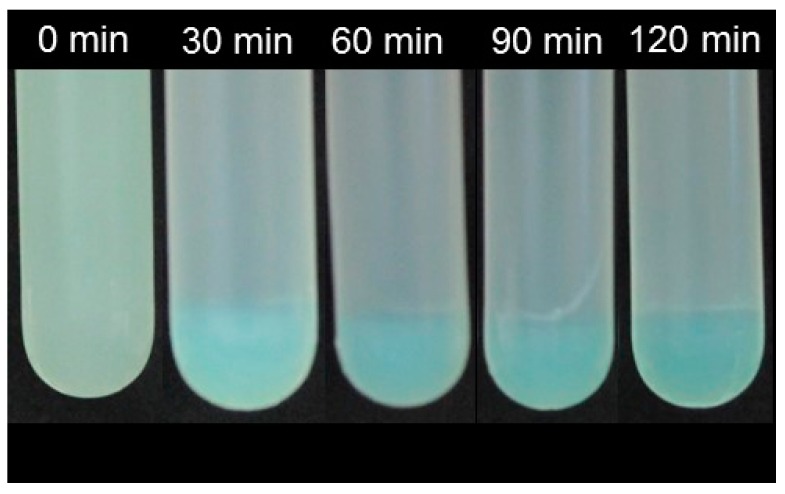
Influence of centrifugation time on the segregation behavior of a mixed protein–polysaccharide system (10 mM phosphate buffer, pH 7): 3.0% caseinate and 1.5% alginate (note: protein phase shown was dyed with methylene blue).

Segregative phase separation has been extensively investigated for mixtures of proteins and polysaccharides [8,9]. These experiments identified the optimum conditions to promote segregation of various types of mixed protein–polysaccharide system. The studies have shown that the precise conditions required depend on the molecular characteristics of both the protein and polysaccharide, such as molecular weight, conformation, hydrophobicity, and charge. We hypothesized that knowledge of the segregative behavior of a model protein–polysaccharide mixture would be useful for analyzing the phase separation of mixtures composed entirely of polysaccharides. For this reason, caseinate (CAS) and alginate (ALG) stock solutions were mixed under neutral conditions (10 mM phosphate buffer, pH 7) at various biopolymer ratios. The resulting mixtures were then centrifuged at 10,000 *g* for up to 120 min to promote phase separation, with aliquots being withdrawn at regular intervals. The segregation of the protein and polysaccharide were visualized by staining the protein with methylene blue (Figure 4).

Segregation of caseinate and alginate were observed at all biopolymer concentrations used, which indicates that all levels used were high enough to promote thermodynamic incompatibility. The photographic images of the samples demonstrated that segregation had occurred within 30 min of centrifugation, but longer centrifugation times led to the formation of a sharper phase boundary between the protein and polysaccharide phase. The methylene blue staining indicated that the lower phase was enriched with caseinate, whereas the upper phase contained mainly alginate molecules. Previous studies have also shown that proteins tend to partition into the lower phase, which can be attributed to the higher density of the protein-rich phase [17]. Based on the study with the protein–polysaccharide combination, we used similar environmental conditions for the polysaccharide–polysaccharide systems.

### 2.5. Mixed Alginate-Beet Pectin Systems

In this section, the segregative phase separation in systems composed of two polysaccharides was studied. We hypothesized that segregation would be promoted in polysaccharide–polysaccharide systems due to a combination of electrostatic, steric exclusion, and hydrophobic forces. Beet pectin (1.0–4.0%) and alginate (1.5–3.0%) were mixed together at pH 7 and then centrifuged for at least 90 min at 10,000 *g* to induce phase separation. The partitioning of each biopolymer was then analyzed using absorbance measurements, calcium sensitivity tests, and pectin staining.

**Figure 5:**
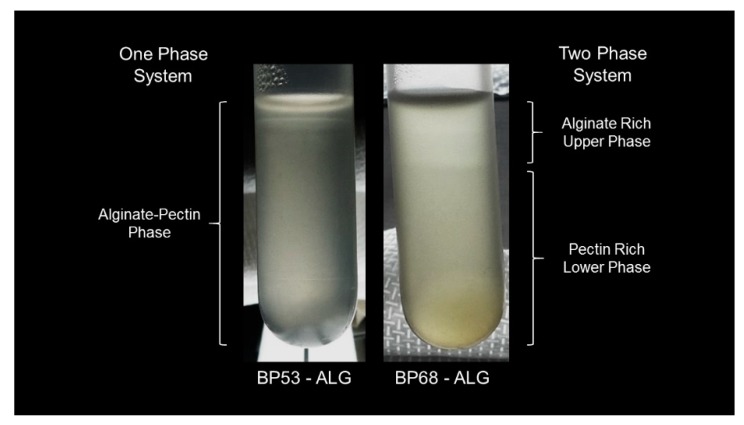
Phase separation behavior of beet pectin–alginate mixtures (10 mM phosphate buffer, pH 7): 2% beet pectin (BP) and 1.5% alginate (ALG).

Our results clearly demonstrated that the degree of polysaccharide hydrophobicity had an impact on the segregation behavior. In general, BP68-ALG mixtures formed a two-phase system regardless of the overall biopolymer concentration (Figure 5). In these systems, the height of the lower phase decreased with increasing pectin concentration. In contrast, no phase separation was observed in the between BP53-ALG mixtures. It was assumed that increasing the number of methyl groups on the backbone of the pectin molecules increased their hydrophobic nature, which promoted more pectin–pectin interactions. Moreover, beet pectin molecules have ferulic acid groups and protein moieties that can also promote pectin–pectin interactions through hydrophobic interactions [30,31].

After segregation, the upper and lower phases were carefully separated to qualitatively determine the pectin and alginate content (Table 1). Both the upper and lower phases gelled after calcium addition, which indicates that some alginate was present in both phases. The lower phase showed a distinct absorbance peak at 325 nm which was attributed to the presence of beet pectin. Sugar beet pectin typically contains high amounts of ferulic acid groups, which are attached to the galactose and arabinose side chains, and absorb in the UV region of the electromagnetic spectrum. In addition, L*a*b measurements revealed a strong red color in the lower phase, which was further evidence that the beet pectin was mostly present in this phase.

## 3. Conclusions

This study demonstrated as a first attempt that mixtures of two different polysaccharides (beet pectin and alginate) could be made to phase separate by modifying the number of methyl groups on the pectin molecules. Increasing the degree of methylation leads to a stronger hydrophobic attraction between the pectin molecules, which presumably promotes pectin–pectin interactions and promotes segregation. Previous studies have shown that segregation in protein–polysaccharide mixtures can be used to create novel structures, textures, and functionalities in foods. Our results suggest that polysaccharide–polysaccharide mixtures may be able to be used for the same purpose. Moreover, this type of binary system can be comprised of two dietary fibers, which may lead to the development of food products with health benefits.

## 4. Materials and Methods

### 4.1. Materials

Sodium caseinate (#L080512201) was purchased from Rovita GmbH (Engelsberg, Germany). Alginic acid sodium salt (#9180.2) was obtained from Carl Roth GmbH & Co. KG. Unmodified and modified high-methoxylated sugar beet pectins (#11408491) were donated by Herbstreith & Fox KG (Neuenbürg, Germany). The physicochemical properties of all the biopolymers used in the study are summarized in Table 2. Calcium chloride (#CN93.2), sodium hydroxide pellets (NaOH), and analytical grade hydrochloric acid (HCl, #P074.4) were purchased from Carl Roth GmbH & Co. KG (Karlsruhe, Germany). Sodium phosphate monobasic monohydrate (#S9638, purity ≥ 98%), hydroxylamine hydrochloride (#55459), iron (III) chloride (#157740), and methylene blue (#M9140) were purchased from Sigma-Aldrich (Steinheim, Germany) or Merck KGaA (Darmstadt, Germany). All biopolymer solutions were centrifuged at 10,000 g for 2 h at 20 °C prior to utilization to remove any insoluble matter. Double distilled water was used in the preparation of all samples.

### 4.2. Experimental Methods

The study was divided into three main parts to observe the phase segregation of polysaccharide–polysaccharide mixtures. First, all biopolymers were electrically, optically, and rheologically analyzed to determine their surface charge, molecular weight, aggregation state, and flow behavior. Second, the characteristics of phase separated systems were determined by measuring the segregation time, phase volumes, and phase composition. Third, alginate–beet pectin mixtures were blended at pH 7, whereas the partitioning of the biopolymers used between the upper and lower phase was determined.

#### 4.2.1. Characterization of Biopolymer Solutions

*ζ*-Potential measurements—A particle electrophoresis instrument (Nano ZS, Malvern Instruments, Malvern, UK) was used to determine the electrical properties (ζ-potential) of the biopolymers in solution. Samples were loaded into an appropriate cuvette and the ζ-potential was determined by measuring the direction and velocity that the particles moved in the applied electric field. The Smoluchowski equation was then used to calculate the ζ-potential. The ζ-potential measurements were made from two freshly prepared samples, and were carried out with four readings per sample.

Turbidity measurements—A UV–visible spectrophotometer (Agilent 8453, Agilent Technologies Inc., Santa Clara, CA, USA) was used to determine the transmission (*T_600 nm_*) of single biopolymer solutions at 600 nm. A measure of the turbidity (%) was then calculated according to Equation (3). Double distilled water was used as a reference.

Turbidity (%) = 100% − *T_600 nm_* (3)

Rheological measurements—The rheological properties of biopolymer suspensions with different concentrations (1.0–44.0% w/w) were investigated using a modular complex rheometer (Physica MCR 502) and associated software (RheoPlus, Anton Paar, Karlsruhe, Germany). The measurements were conducted at 25 °C using a single gap cylinder geometry (CC27 with 28.92 mm cap diameter and 26.66 mm bob diameter, Anton Paar, Karlsruhe, Germany). Flow curves of the solutions were determined by recording the shear stress (τ) at increasing shear rates ($\dot{\gamma }$) from 0.00001 s^−1^ to 100 s^−1^ at 40 measurement points on a time interval of 400 s.

#### 4.2.2. Preparation and Characterization of Mixed Biopolymer Systems

Caseinate–alginate system (reference system)—A mixture of caseinate and alginate was used as a reference system because it is known that segregative separation occurs for these two biopolymers under neutral pH conditions [32,33]. In brief, caseinate and alginate stock solutions were blended together at a ratio of 1:1 (total volume 50 mL) using a magnetic stirrer for at least 1 hour. Mixed solutions with various protein concentrations (1.0–3.0% w/w) and alginate concentrations (0.75–1.0% w/w) were prepared in buffer solution (10 mM sodium phosphate, pH 7). The protein fraction was stained using methylene blue to better visualize the partitioning. After mixing, the system was re-adjusted to pH 7 using 0.1 and 1 M HCl and/or NaOH solutions. Subsequently, 20 g of the mixtures were loaded into centrifugation tubes and centrifuged at 10,000 g for 120 min at 20°C. At regular time intervals, aliquots were withdrawn to visually observe the segregation between the caseinate and alginate mixture using a digital camera (PowerShot G10, Canon, Arlington, VA, USA).

Beet pectin–alginate system—Polysaccharide–polysaccharide mixtures composed of beet pectin and alginate were prepared using the same methods. The phase separated systems were divided to qualitatively determine the partitioning of each biopolymer in the upper or lower phase after centrifugation.

Photometric absorbance measurements—Beet pectins show a distinct absorbance peak at 325 nm due to the presence of ferulic acid moieties [22]. Therefore, the biopolymer solutions were placed in a cuvette followed by measuring the absorbance at 325 nm at 25 °C using a UV–visible light spectrophotometer (HP 8453, Agilent with application software Chemstation Agilent Technologies 95-00, Waldbronn, Germany).

Pectin staining—An established staining approach was used to visualize the pectin in the samples [34,35]. In brief, pectin samples were initially hydrated in 10 mM phosphate buffer (pH 7), whereas alkaline hydroxylamine reagent was prepared according to Reeve (1959) and Hornatowska (2005). Subsequently, equal weights of alkaline hydroxylamine reagent, concentrated HCl, and 10% ferric chloride solution were mixed with the biopolymer phases followed by incubation at room temperature for 7 min. After incubation, the change in color was determined by a colorimeter (Chroma meter CR-200, Minolta, Tokio, Japan).

Calcium sensitivity test—The sensitivity of the alginates towards multivalent ions is known to be high, which can be used to detect their presence [29,36]. Hence, 20 µL of a 10 mM CaCl_2_ stock solution was added to both the upper and lower biopolymer phase to promote gelation. Subsequently, the test tubes were turned upside down to determine which phase had gelled (and was therefore rich in alginate). An immediate gelation of alginate was defined as a high sensitivity level.

#### 4.2.3. Statistical Analysis

All experiments were repeated at least twice using freshly prepared samples, with repeated measurements being made for each sample. Means and standard deviations were calculated from a minimum of three measurements using Excel. Results were analyzed using a statistical software (SigmaPlot 12.5, Systat Software, Inc., San Jose, CA, USA).

## Figures and Tables

**Table 1:** Characterization of the upper and lower biopolymer phase of a mixed beet pectin–alginate system (10 mM phosphate buffer, pH 7)

Phase	Absorbance at λ = 325 nm	a*−value	Calcium Sensitivity ^1^
Upper	0.75 ± 0.00	−0.15 ± 0.04	++
Lower	1.04 ± 0.01	1.56 ± 0.13	+
Alginate	0.04 ± 0.00	n.d.	+++
Beet pectin (DE 53%)	1.11 ± 0.01	n.d.	–

^1^ +++ = high sensitivity; + = low sensitivity; – = no gelation.

**Table 2:** Physicochemical properties of the biopolymers used to investigate the segregative phase separation

Biopolymer	Abbreviation	Characteristics ^1^
Sodium caseinate	CAS	≥ 88% protein; ≤ 6% moisture; ≤ 4.5% minerals; ≤ 0.2% fat; ≤ 0.2% lactose
Alginate	ALG	Guluronic acid:mannuronic acid = 0.75 ± 0.02
Sugar beet pectin	BP53	DE° = 53.4%; DAc° = 25.8%; AGA = 65.4%; ferulic acid = 0.75 ± 0.02%; pH = 2.82
Modified sugar beet pectin	BP68	DE° = 68.3%; DAc° = 20.4%; AGA = 70.0%; pH = 4.70

^1^ DE° = degree of esterification; DAc° = degree of acetylation; AGA = anhydrous galacturonic acid.

## Supplementary Materials

The following are available online at https://www.mdpi.com/2310-2861/5/2/26/s1.
