# Supplementary material for: Segregation Behavior of Polysaccharide–Polysaccharide Mixtures—A Feasibility Study
Source: Gels. 2019 May 13;5(2):26. doi: 10.3390/gels5020026 (PMC6631692; doi:10.3390/gels5020026)
Supplement: Supplementary file 1 [file gels-05-00026-s001.pdf]

Segregation Behavior of Polysaccharide–Polysaccharide Mixtures—A Feasibility Study

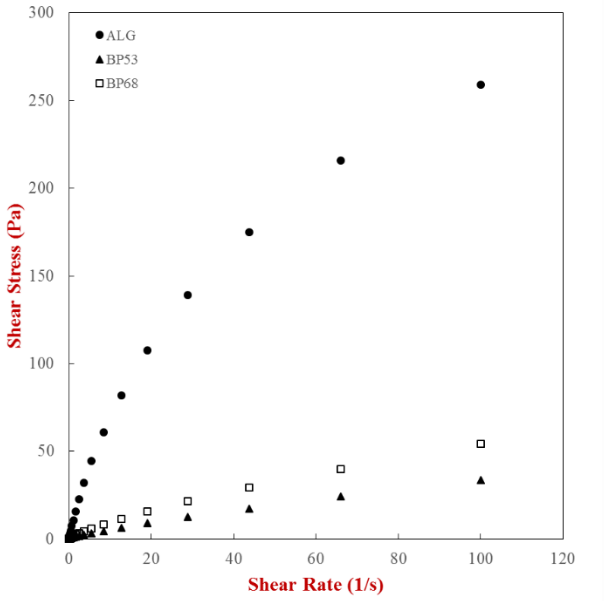

Figure S1. Shear thinning behavior of single biopolymer solutions ($c_{biopolymer} = 4$ %): Alginate (ALG), sugar beet pectin DE 53% (BP53), and sugar beet pectin DE 68% (BP68).
